# Subcellular Heterogeneity of Ryanodine Receptor Properties in Ventricular Myocytes with Low T-Tubule Density

**DOI:** 10.1371/journal.pone.0025100

**Published:** 2011-10-13

**Authors:** Liesbeth Biesmans, Niall Macquaide, Frank R. Heinzel, Virginie Bito, Godfrey L. Smith, Karin R. Sipido

**Affiliations:** 1 Laboratory of Experimental Cardiology, University of Leuven, Leuven, Belgium; 2 Faculty of Biomedical and Life Sciences, University of Glasgow, Glasgow, United Kingdom; 3 Division of Cardiology, Medical University of Graz, Graz, Austria; Brigham & Women's Hospital - Harvard Medical School, United States of America

## Abstract

**Rationale:**

In ventricular myocytes of large mammals, not all ryanodine receptor (RyR) clusters are associated with T-tubules (TTs); this fraction increases with cellular remodeling after myocardial infarction (MI).

**Objective:**

To characterize RyR functional properties in relation to TT proximity, at baseline and after MI.

**Methods:**

Myocytes were isolated from left ventricle of healthy pigs (CTRL) or from the area adjacent to a myocardial infarction (MI). Ca^2+^ transients were measured under whole-cell voltage clamp during confocal linescan imaging (fluo-3) and segmented according to proximity of TTs (sites of early Ca^2+^ release, F>F_50_ within 20 ms) or their absence (delayed areas). Spontaneous Ca^2+^ release events during diastole, Ca^2+^ sparks, reflecting RyR activity and properties, were subsequently assigned to either category.

**Results:**

In CTRL, spark frequency was higher in proximity of TTs, but spark duration was significantly shorter. Block of Na^+^/Ca^2+^ exchanger (NCX) prolonged spark duration selectively near TTs, while block of Ca^2+^ influx via Ca^2+^ channels did not affect sparks properties. In MI, total spark mass was increased in line with higher SR Ca^2+^ content. Extremely long sparks (>47.6 ms) occurred more frequently. The fraction of near-TT sparks was reduced; frequency increased mainly in delayed sites. Increased duration was seen in near-TT sparks only; Ca^2+^ removal by NCX at the membrane was significantly lower in MI.

**Conclusion:**

TT proximity modulates RyR cluster properties resulting in intracellular heterogeneity of diastolic spark activity. Remodeling in the area adjacent to MI differentially affects these RyR subpopulations. Reduction of the number of sparks near TTs and reduced local NCX removal limit cellular Ca^2+^ loss and raise SR Ca^2+^ content, but may promote Ca^2+^ waves.

## Introduction

In ventricular cardiac myocytes, Ca^2+^ influx through voltage-gated L-type Ca^2+^ channels (LTCC) activates Ca^2+^ release from ryanodine receptors (RyRs) on the sarcoplasmic reticulum (SR). This Ca^2+^-induced Ca^2+^ release occurs in local release units or couplons, i.e. clusters of LTCC facing clusters of RyR, which are preferentially located in the T-tubules (TT). The preferential location of couplons in TTs [1], [2] make TTs critically important for the synchrony of Ca^2+^ release. Indeed, the [Ca^2+^]_i_ transient results from spatiotemporal summation of elementary release events, or Ca^2+^ sparks [3], [4], which, in rat ventricular myocytes, are mainly localized near TTs [5], [6]. Sparks can also occur during diastole, representing spontaneous probabilistic activation of a RyR or a RyR cluster [3].

TTs are poorly developed or absent in neonatal myocytes and in cells from the specialized conduction system such as Purkinje cells, as well as in atrial cells. These cells are small and show a radial spread of Ca^2+^ from the external sarcolemma to the center mediated by a combination of Ca^2+^ release and diffusion [7], [8]. Experimental disruption of TTs in ventricular myocytes by osmotic shock or culture has been shown to reduce the synchrony of Ca^2+^ release, and depress and slow the Ca^2+^ transient [9]–[11]. In pig ventricular myocytes, as in human myocytes, TTs are less developed than in rodents [10], [12], [13]. In contrast, RyRs are distributed homogeneously and regularly throughout the cells, indicating that there is relatively more non-junctional RyRs than in ventricular myocytes of rodents. Ca^2+^ release during excitation-contraction coupling is consequently less synchronized and areas of delayed Ca^2+^ release were shown to result from local absence of TTs [12]. This study did not show intrinsic differences of RyR related to TT presence, but analysis was limited to the globally triggered release, which may mask such differences.

Ca^2+^ sparks, reflecting intrinsic activity and properties of RyR clusters, may provide more information. In ventricular myocytes these mainly occur at the junctional SR [14] while in atrial cells lacking TTs, sparks occur near the sarcolemma but also in the central areas. Studies in cat atrial cells showed a higher frequency of near-sarcolemmal junctional sparks than of central non-junctional sparks, while amplitude, width and duration were comparable [15], although in rat atrial myocytes the time course of subsarcolemmal sparks was shorter and with a reduced local spread [16]. Data informing whether in ventricular myocytes a sparse TT network could lead to inhomogeneities of RyR cluster properties are limited. In ventricular myocytes from dogs with heart failure, 2D imaging showed non-uniform distribution of sparks related to loss of TTs with less activity in areas devoid of TTs [17].

In the present study, we build on our earlier data where we identified areas without TTs within pig ventricular myocytes based on the local Ca^2+^ transient properties during normally triggered SR Ca^2+^ release [12], [18]. We use this approach as a tool to assess the effects of TT proximity on spontaneous spark and RyR properties during diastole. We further examine RyR properties in a pig model of ischemic cardiomyopathy, where TT density was significantly reduced [18]. The data show the presence of significant heterogeneity of sparks at baseline with further modulation after remodeling in the area adjacent to a myocardial infarction (MI).

## Materials and Methods

### Pig model of ischemic cardiomyopathy

Animals were housed and treated according to the Guide for the Care and Use of Laboratory Animals (National Institute of Health, U.S.A.) and experimental protocols were approved by the in-house ethical committee (*Ethische Commissie Dierproeven*, Katholieke Universiteit Leuven), with permit numbers P02042, P06048 and P10139.

The MI data in the current study are derived from the same pigs described earlier [18]. All interventions were performed under full anesthesia (premedication with tiletamine and zolazepam, 4 mg/kg IM and xylazine 0.25 mg/kg IM, maintenance anesthesia with propofol IV, 7 mg/kg/hour; additional analgesia with buprenorphine HCl 0.1 mg/kg). Implantation of a copper-coated stent in the proximal circumflex coronary artery of young adult domestic pigs (20–25 kg) induced intima proliferation and a high degree stenosis (>90%) within 10–15 days. After 6 weeks, global LV function and extent of MI were evaluated with magnetic resonance imaging; included pigs had only modest reduction of the ejection fraction with an MI that extended on average less than 10% of the LV mass [18]. Pigs were sacrificed 24–48 hrs later (N_pigs_ MI = 7) and compared to healthy weight-matched controls (N_pigs_ CTRL = 12). To evaluate the effect of sarcolemmal fluxes and culture, additional control pigs were used in the present study (N_pigs_ = 12).

### Isolation of cardiomyocytes

Cardiac myocytes were isolated enzymatically as described previously [18]. Briefly, the coronary artery was cannulated distal to the stenosis and the tissue wedge perfused on a Langendorf setup at 37°C. After perfusion with low Ca^2+^ Tyrode (in mmol/L: NaCl 130, KCl 5.4, KH_2_PO_4_ 1.2, MgSO_4_ 1.2, CaCl_2_ 0.18, Na-Hepes 6, glucose 20, pH 7.2), the tissue was perfused with 1.4 g/L, collagenase A (Roche, Germany) and 0.1 g/L,protease XIV, (Sigma-Aldrich, Belgium), followed by washout with low Ca^2+^ tyrode. After removal of the tissue wedge from the setup, the infarct area and border zone were discarded and cells were obtained by gentle trituration of the digested midmyocardial tissue adjacent to the MI. The obtained cell suspension was filtered and resuspended in normal Tyrode (in mmol/L, NaCl 137, KCl 5.4, MgCl_2_ 0.5, CaCl_2_ 1.8, Na-HEPES 11.8, and glucose 10; pH 7.4). CTRL cells were isolated from the same area of matched healthy pigs.

In a number of experiments, freshly isolated cardiomyocytes from CTRL pigs were put in primary culture for 48 h as described previously [10]. Cultured cells were compared with freshly isolated cells from the same pigs (N_pigs_ CTRL = 7).

### Immunofluorescence

Myocytes were fixed with 2% paraformaldehyde and permeabilised with 0.5% Triton X-100 in PBS. They were washed 3 times and incubated overnight with IgG-anti-RyR primary antibody (clone 34C, 1/100 in PBS, Affinity BioReagents, Golden, CO, USA) at 4°C. The primary antibody was rinsed with PBS and the secondary antibody, FITC-conjugated anti-mouse IgG, was added to the fixed myocytes for 2 hours (1/250 in PBS, Sigma-Aldrich, Belgium).

### Electrophysiology and global [Ca^2+^]_i_ measurements

Transmembrane ionic currents were recorded using an Axon 200B amplifier using whole-cell voltage clamp in the ruptured patch configuration. Pipette solution contained (in mmol/L: K-aspartate 120, NaCl 10, KCl 20, K-HEPES 10, MgATP 5, and K_5_fluo-3 0.05; pH 7.2). Inclusion of fluo-3 salt in the pipette solution allowed simultaneous measurements of ionic currents and [Ca^2+^]_i_ transients. Patch pipettes (GB 200-8P, Science products) had a resistance of 2.0–3.0 MΩ. All experiments were carried out at 37°C in normal Tyrode solution. Where indicated NiCl_2_ (5 mmol/L) or CdCl_2_ (50 µmol/L) were added to this solution and applied through a fast perfusion system. Experiments with Ni^2+^ were performed in the presence of 1 µmol/L forskolin.

[Ca^2+^]_i_ transients were elicited by depolarizing pulses (250 ms) from −70 to +10 mV at 1 Hz. NCX current density was measured as the inward tail current 10 ms after repolarization. SR Ca^2+^ content was measured by integrating the inward Na^+^/Ca^2+^ exchange (NCX) current during fast caffeine application (10 mmol/L) and expressed in terms of accessible cell volume.

### Confocal microscopy

Spatiotemporal characteristics of [Ca^2+^]_i_ transients and spontaneous Ca^2+^ sparks were studied using a Zeiss Axiovert 100 M inverted microscope with a ×40/1.3 oil-immersion objective in combination with a Zeiss LSM 510 confocal laser point-scanning system (LSM 510, Zeiss, Jena, Germany). Quiescent, rod-shaped myocytes with clear striations were randomly selected. The 488 nm line of a 25 mW argon laser was used for excitation of Fluo-3. Ca^2+^ release was visualized by repetitive scanning of a line selected longitudinally through the cell, avoiding the nuclei. X and Y resolution were 0.2 to 0.4 µm and 1.54 ms respectively. Stimulated Ca^2+^ transients were recorded after reaching a steady state. Stimulation was subsequently stopped and spontaneous Ca^2+^ sparks were recorded for 15 s.

### Image analysis

Linescan images of [Ca^2+^]_i_ transients and spontaneous Ca^2+^ sparks were analyzed using a custom-made program, modified after [19]. Fluorescence was normalized to the diastolic [Ca^2+^]_i_ level (F/F_0_). Half-maximal Ca^2+^ release (F_50_) of the averaged [Ca^2+^]_i_ transient served as reference and threshold for categorizing local Ca^2+^ release areas into early and late release areas, respectively near-TTs and more remote. This correlation with the TT network was established earlier [12], and confirmed in the same animal population used in the present study [18]. Early release areas are those that reached F_50_ within 20 ms after the start of the [Ca^2+^]_i_ transient; delayed release areas are those that did not.

Spontaneous Ca^2+^ sparks were detected automatically, based on [20] (Cri 4.5). Sparks were assigned to bins of 3 µm width across the scan line. Each bin was linked to the local release area in the averaged [Ca^2+^]_i_ transient in the corresponding part of the scan line and thus sparks were categorized as occurring near TTs (early release areas) or more remote. Spark properties were subsequently analyzed per category, i.e. associated with early or delayed release sites; spark frequency was likewise normalized within each category, i.e. number of events per area length with early or delayed release sites. Spark width and duration are shown as Full-Width at Half-Maximum (FWHM) and Full-Duration at Half-Maximum (FDHM) respectively. Sparks were considered as extremely long when more than three standard deviations above the average spark duration in CTRL (47.6 ms).

### Statistics

Data are shown as mean ± SEM, averaged by animal. Comparisons were done using Student's t-test, or Mann Whitney test, as appropriate. P<0.05 was considered significant.

## Results

### Heterogeneous spark properties in pig ventricular myocytes

**Figure 1:**
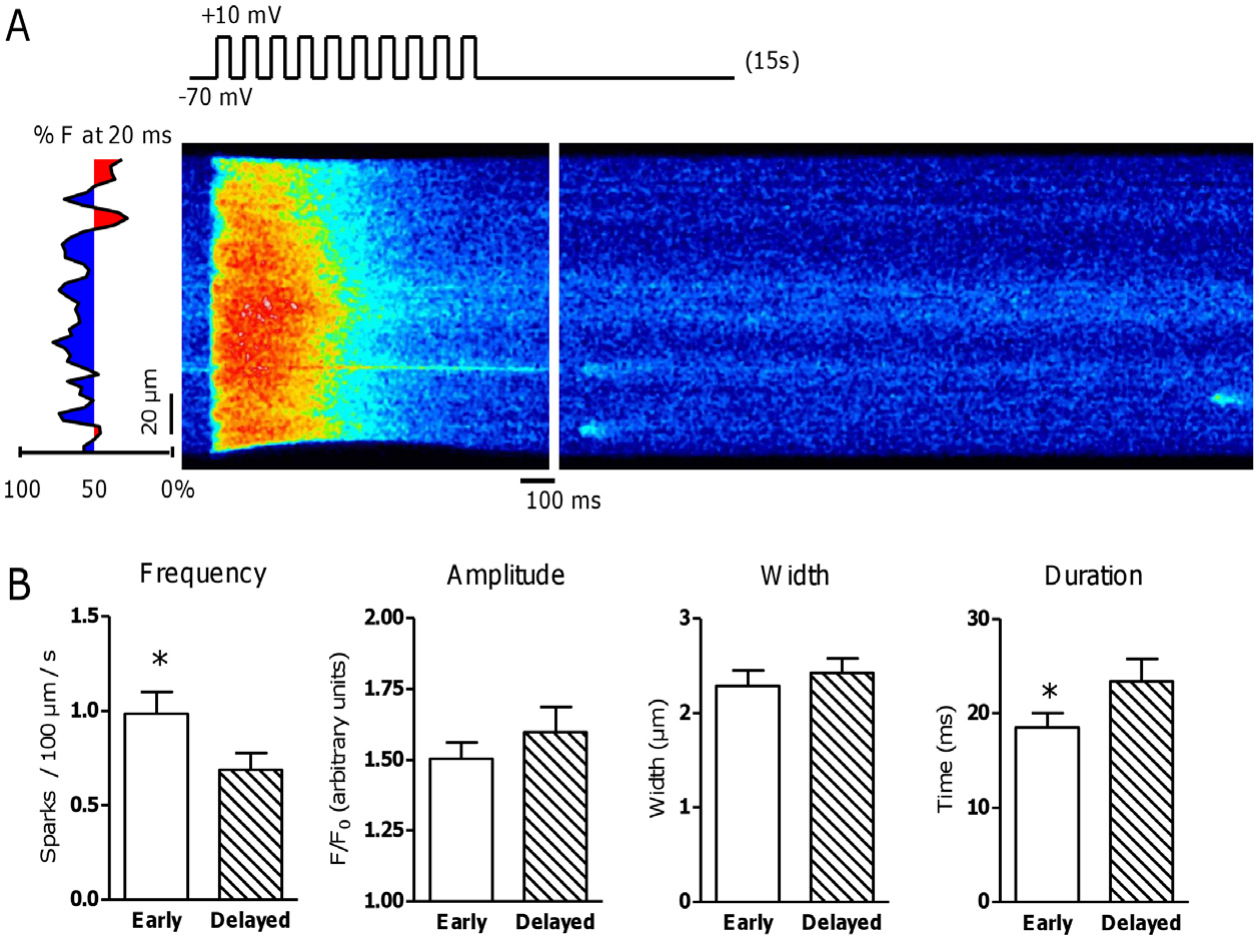
Effect of proximity of TTs to RyR on spontaneous Ca^2+^ sparks. A. Typical example of a line scan image during and after 1 Hz stimulation. After loading the SR with conditioning pulses from −70 to +10 mV at 1 Hz, stimulation was stopped and 15 seconds of diastole were recorded for Ca^2+^ sparks. Sparks were assigned to early (blue) and delayed (red) release areas corresponding to their position on the scan line. B. Spark frequency and morphology in early vs. delayed release areas in CTRL pigs (N_pigs_ = 12, n_cells_ = 41). * denotes P<0.05.

Spontaneously occurring Ca^2+^ sparks were recorded after stimulation at 1 Hz to load the SR to steady state and assigned to early or delayed release areas according to their position on the scan line, as shown in Fig. 1A. In CTRL myocytes, spark frequency was significantly higher in early than in delayed release areas (Fig. 1B). Spark amplitude and width were comparable (Fig. 1B). Spark duration was significantly shorter in early than in delayed release areas (Fig. 1B).

### The role of sarcolemmal fluxes in spark frequency and duration

**Figure 2:**
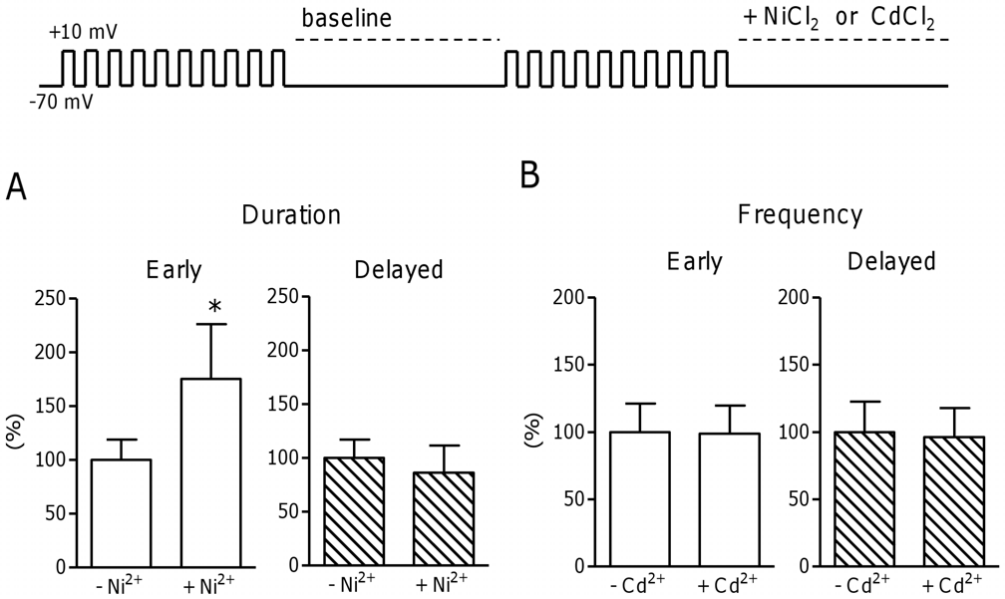
Effect of sarcolemmal fluxes on spark frequency and duration. A. Effect of NCX block by 5 mM nickel on spark frequency and duration in early (left) and delayed (right) release areas in CTRL cardiomyocytes (N_pigs_ = 3, n_cells_ = 7). B. Effect of I_CaL_ block by 50 µM cadmium on spark frequency and duration in early (left) and delayed (right) release areas in CTRL cardiomyocytes (N_pigs_ = 2, n_cells_ = 9). * denotes P<0.05.

Since NCX is known to be highly expressed in TTs [2], [21], we investigated whether NCX affects spark duration preferentially in early release areas. Therefore we compared spark duration with and without NCX block (Ni^2+^, 5 mmol/L NiCl_2_) applied for the duration of the spark recording, as shown in the inset of Fig. 2. Spark duration was significantly prolonged with Ni^2+^ in early, but not delayed release areas (Fig. 2A). Yet block of NCX did not alter spark frequency, nor baseline fluorescence (data not shown). Spark amplitude and width were also unchanged (data not shown).

As Ni^2+^ also blocks Ca^2+^ entry through LTCC, the data suggest Ca^2+^ entry through LTCC does not influence spark frequency. This was further examined more specifically by comparing sparks with and without LTCC block by Cd^2+^ (50 µM CdCl_2_). I_CaL_ inhibition indeed did not affect spark frequency in early or delayed release areas (Fig. 2B). Spark morphology was also unchanged (data not shown).

### The effect of remodeling in the area adjacent to MI on Ca^2+^ sparks

**Figure 3:**
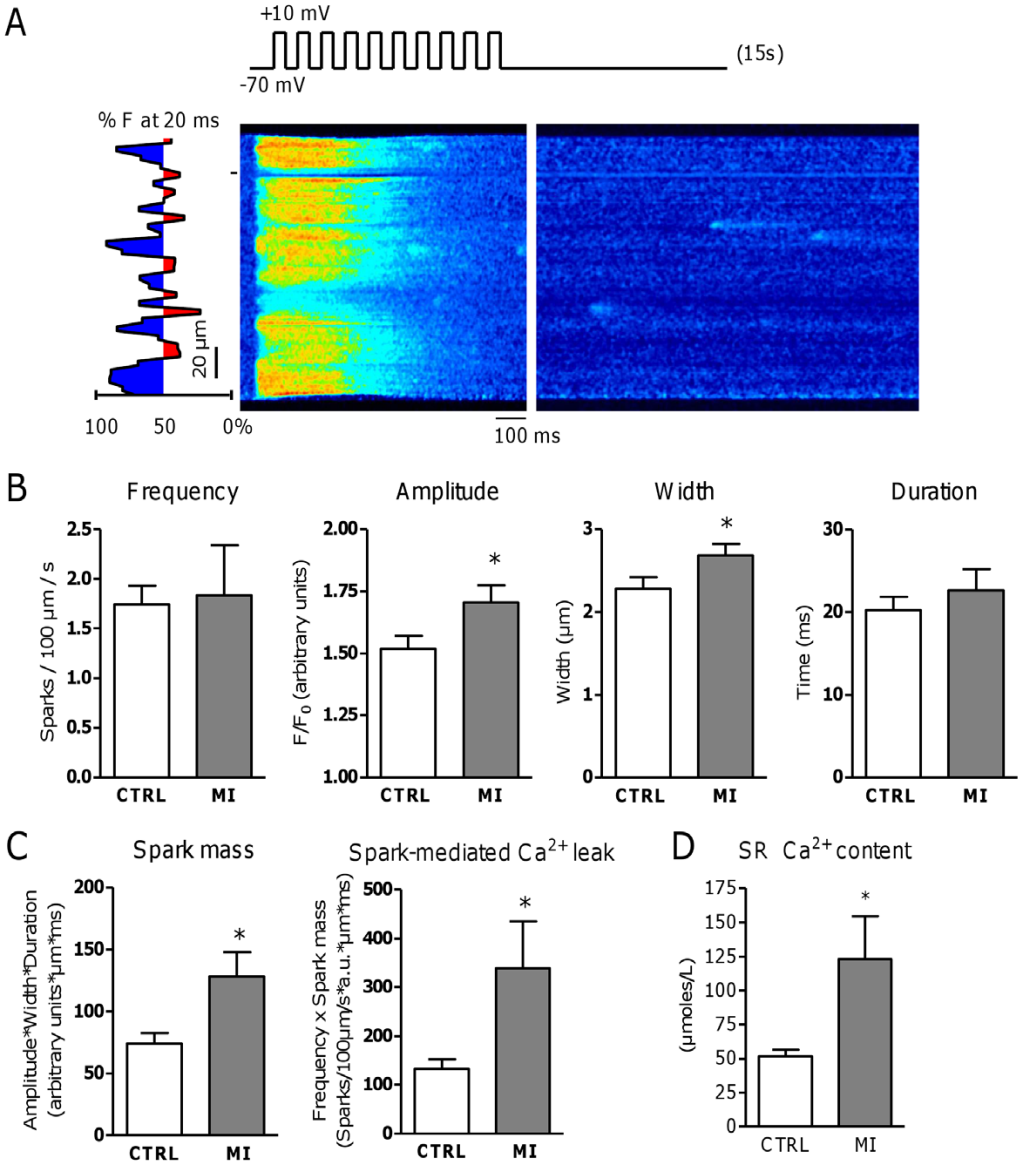
Effect of remodeling in the area adjacent to MI on Ca^2+^ sparks. A. Representative line scan image of Ca^2+^ spark recording in MI. The types of release areas are marked in blue and red for early and delayed release areas respectively. The artifact region (marked by –) was excluded for analysis. B. Whole-cell spark frequency and morphology in CTRL (N_pigs_ = 12, n_cells_ = 41) and MI (N_pigs_ = 7, n_cells_ = 33). C. Spark mass, calculated by amplitude*width*duration, and spark-mediated Ca^2+^ leak, calculated by spark frequency*spark mass, in CTRL (N_pigs_ = 12, n_cells_ = 41) and MI (N_pigs_ = 7, n_cells_ = 33). D. SR Ca^2+^ content as µmoles/L accessible cytosol, in CTRL (N_pigs_ = 4, n_cells_ = 15) and MI (N_pigs_ = 4, n_cells_ = 12). * denotes P<0.05.

We first examined the properties of the total of Ca^2+^ sparks as is usually reported, i.e. without regional analysis. Average spark frequency in MI myocytes was not different from CTRL but spark amplitude and width were significantly increased in MI; average spark duration was not different (Fig. 3B). Spark mass, calculated as spark amplitude*width*duration, was significantly higher in MI than in CTRL and the spark-mediated Ca^2+^ leak from the SR, defined as spark frequency*spark mass, was thus also increased significantly in MI, by nearly three-fold (Fig. 3C). These data must be viewed against the SR Ca^2+^ content which is a main modulator of leak [22]. SR Ca^2+^ content was calculated from caffeine-induced Ca^2+^ release and the accompanying NCX current [18]. Taking into account relative changes in cell content, SR Ca^2+^ content was significantly higher in MI (123±32 µmoles/L vs. was 52±5 µmoles/L in CTRL, P<0.05; Fig. 3D).

### Differential remodeling of spark properties in MI

Subsequently, we compared sparks in early and delayed release areas within the MI group. As reported before, due to the decrease in TT density [18], the number and extent of delayed release areas was larger in MI. The fraction of sparks in early areas was reduced (50.6±6.3% in MI, N_pigs_ = 7, n_cells_ = 33, *vs.* 60.4±3.2% in CTRL, N_pigs_ = 12, n_cells_ = 41, P<0.05). We next examined spark parameters which in CTRL were different between early and delayed sites.

**Figure 4:**
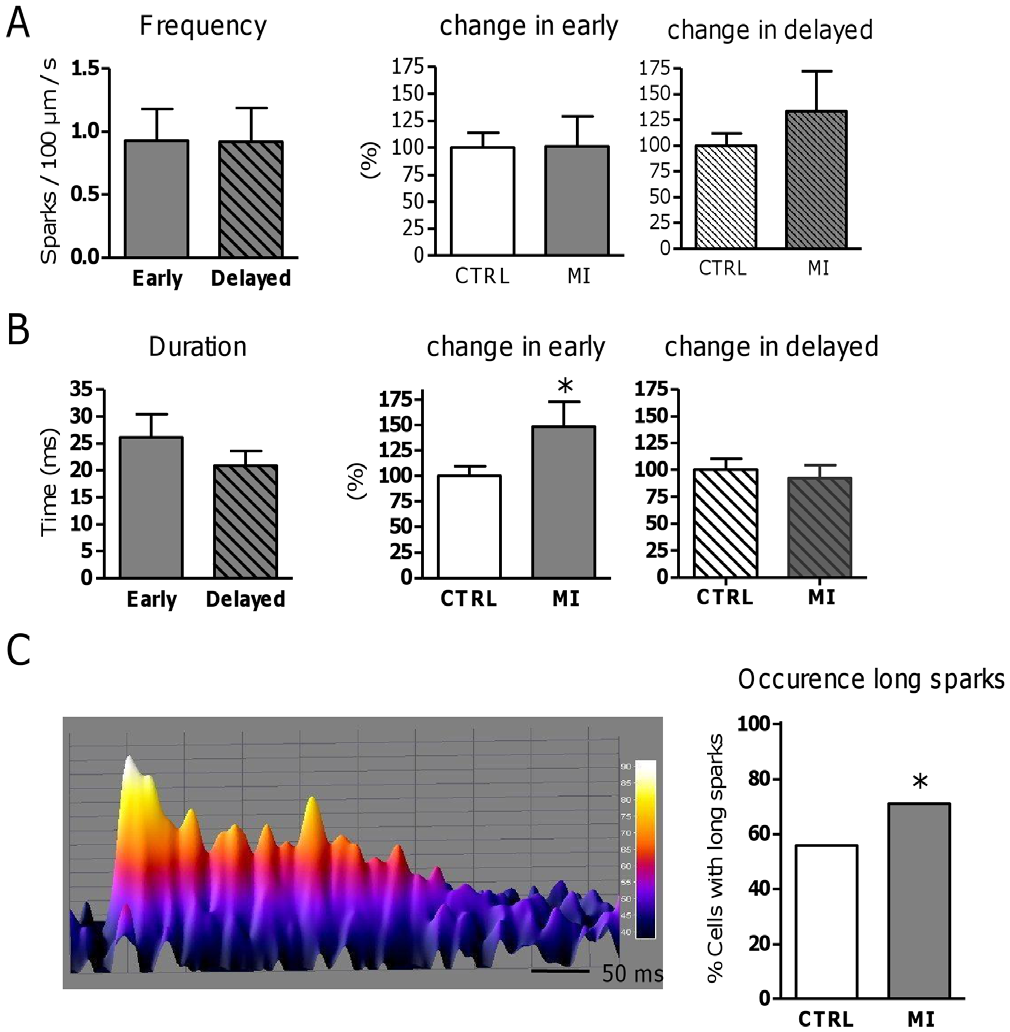
Subcellular spark properties in myocytes from the area adjacent to MI. A. Spark frequency, and, B, duration in early vs. delayed release areas in MI, with the MI-dependent change in each area (CTRL N_pigs_ = 12, n_cells_ = 41 vs. MI N_pigs_ = 7, n_cells_ = 33). C. Example of long spark in 3D and fraction of cells showing long-lasting sparks (>47.6 ms) in CTRL (24/43 cells with long sparks) and MI (22/31 cells with long sparks). * denotes P<0.05.

In contrast to CTRL, no relation between spark frequency and the type of release area was found in MI (Fig. 4A). This was rather due to an increase in spark frequency in delayed areas than to changes in early areas.

In contrast to CTRL animals, spark duration was not different between early and delayed release areas in MI (Fig. 4B). This was due to prolonged spark duration in early areas in MI but not in delayed areas. We also found extremely long sparks (Fig. 4C), categorized as sparks with a duration three standard deviations above the average spark duration in CTRL. The lower threshold for these long sparks was 47.6 ms, but many were found to be longer than 100 ms. The percentage of cells showing long sparks was significantly higher in MI than in CTRL.

### Reduced NCX removal at the sarcolemma in MI

**Figure 5:**
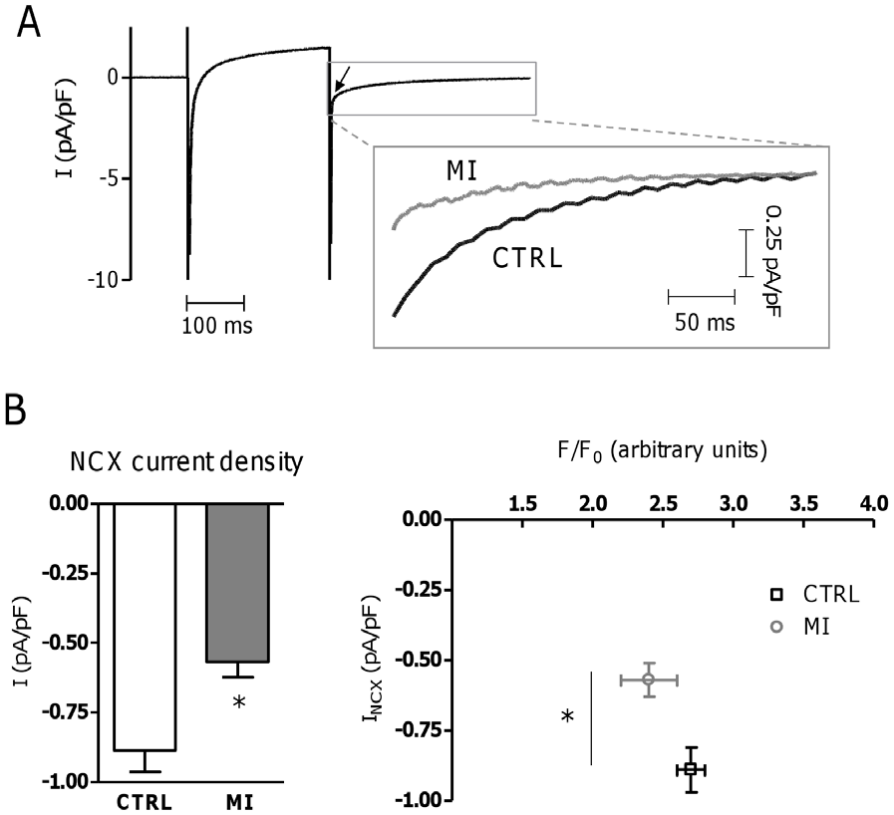
NCX in myocytes from the area adjacent to MI. A. NCX function was measured as the tail current upon repolarization to −70 mV (arrow) after a step to +10 mV in CTRL and MI. B. Averaged NCX current density in CTRL (N_pigs_ = 9, n_cells_ = 30) and MI (N_pigs_ = 7, n_cells_ = 19), with [Ca^2+^]_i_ measured simultaneously. * denotes P<0.05.

With spark duration prolonged in early, but not delayed areas in MI, we hypothesized that changes in NCX function in MI were responsible for this selective prolongation of near-TT sparks. We had previously reported that global Ca^2+^ removal by NCX from myocytes was reduced in MI but this could be related to the reduced surface area with loss of TTs; protein expression levels were unchanged [18]. Now we examined removal per surface area as the inward NCX current density on repolarization (Fig. 5B). This was significantly lower in MI than in CTRL, while [Ca^2+^]_i_ measured at the same time point was comparable (Fig. 5C).

### Cell culture as a model for extensive loss of TTs

**Figure 6:**
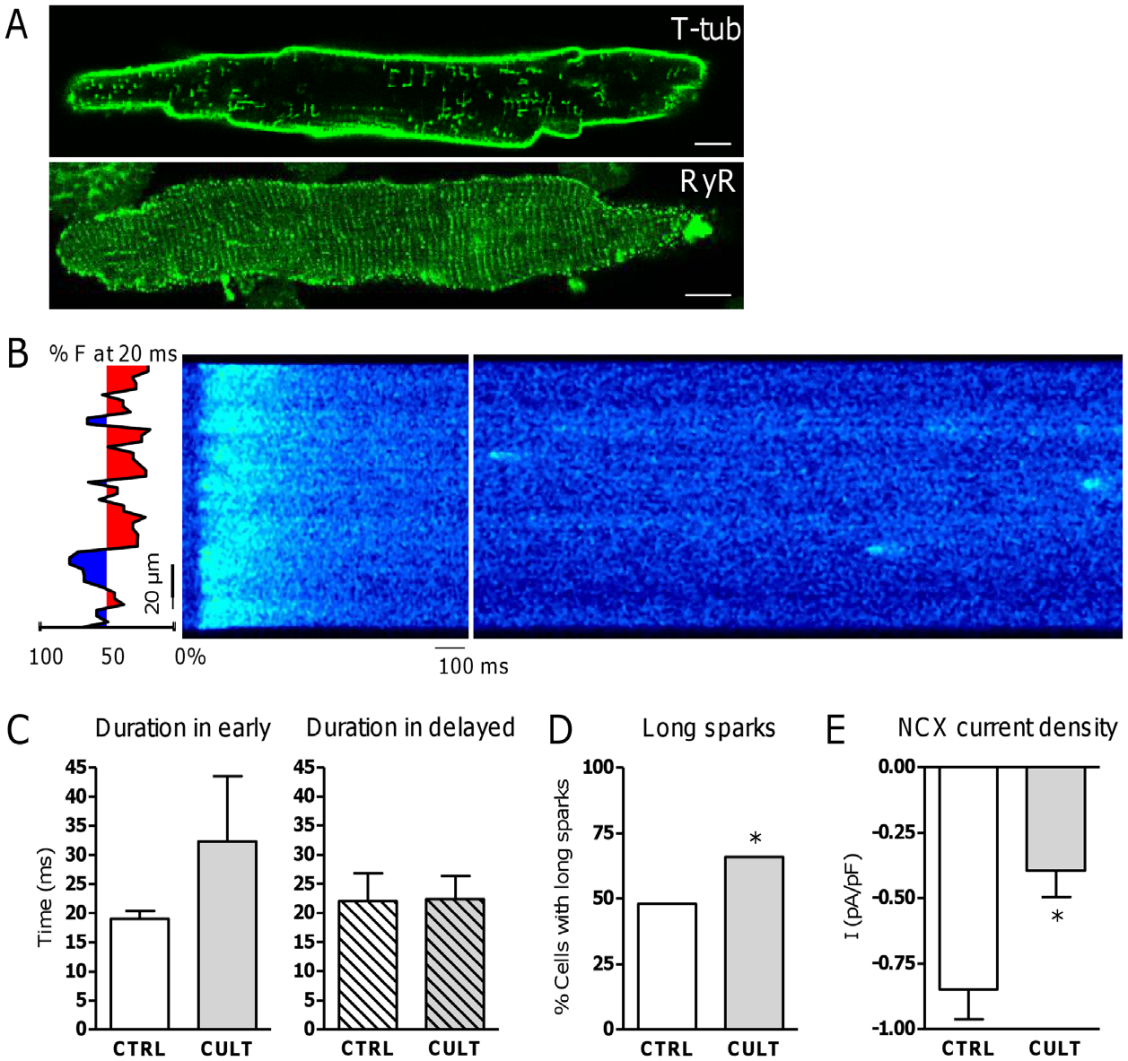
Modulation of Ca^2+^ sparks with TT loss during culture. A. Representative confocal images of TT staining with di-8-ANEPPS (left) and RyR staining (right) after 48 hours of culture. B. Representative line scan image of Ca^2+^ spark protocol in CULT. The type of release areas is marked in blue and red for early and delayed release areas respectively. C. Comparison of spark duration in early (left) and delayed (right) release areas in CTRL (N_pigs_ = 7, n_cells_ = 29) vs. CULT (N_pigs_ = 7, n_cells_ = 14). D. Fraction of cells showing long-lasting sparks (>47.6 ms) in CTRL (17/35 cells with long sparks) and CULT (10/15 cells with long sparks). E. NCX current density in CTRL (N_pigs_ = 4, n_cells_ = 14) and CULT (N_pigs_ = 4, n_cells_ = 7). * denotes P<0.05.

To assess the effect of TT loss on RyR function, we studied myocytes cultured for 48 hours (Fig. 6A). As expected, TT density was decreased significantly but, like in MI, RyR density and distribution remained unchanged (Fig. 6A). The fraction of delayed release areas was increased by 31% in culture (P<0.05). On average spark frequency and morphology were comparable between CULT and CTRL (data not shown). Spark mass and calculated leak from sparks was likewise not different and SR Ca^2+^ content was unchanged in culture (70±12 µmoles/L in CULT, N_pigs_ = 6, n_cells_ = 19, *vs.* 76±9 µmoles/L in CTRL, N_pigs_ = 6, n_cells_ = 16, P = NS). Analysis per region (Fig. 6B) showed that spark duration was not different between early and delayed release areas (32.9±11.3 ms in early *vs.* 22.4±4.0 ms in delayed, N_pigs_ = 7, n_cells_ = 14, P = NS). Compared to CTRL cells, spark duration tended to increase selectively in early release areas (Fig. 6C). The percentage of cells showing extremely long sparks was also significantly increased compared to CTRL (Fig. 6D). NCX current density was significantly decreased in culture, however, [Ca^2+^]_i_ at that time point also tended to be lower (Fig. 6E).

Taken together the data in CULT are close to what is observed in MI, but there are also differences, such as maintained SR Ca^2+^ content and less reduction of NCX compared to the MI group, suggesting these occur as independent events in remodeling.

## Discussion

### Heterogeneity of spark frequency in normal pig ventricular myocytes

Spontaneous spark frequency was higher in early release sites implying that proximity of TTs increases RyR P_0_. In rat ventricular myocytes, spontaneous Ca^2+^ spark frequency was similar throughout intact cells, but detubulation decreased spark frequency in the central regions of the cells [11], [23]. In dog ventricular myocytes with a lower TT density, spontaneous sparks colocalized with TTs [17], consistent with the results of the present study. In rat atrial myocytes, there is also a higher propensity for spontaneous Ca^2+^ sparks in the junctional SR [24].

One possible mechanism for a higher spark frequency near TTs is background Ca^2+^ entry through L-type Ca^2+^ channels. However, block of I_CaL_ did not affect spark frequency in early release areas. This is in agreement with earlier studies showing that I_CaL_ block or removal of extracellular Ca^2+^ did not affect spark frequency in rat ventricular myocytes, indicating sparks were indeed ‘spontaneous’ and did not require sarcolemmal Ca^2+^ influx. In atrial myocytes from the cat, Ca^2+^ influx was important for the higher spark frequency near the subsarcolemma [15], but in rat atrial myocytes it was not [25]. The latter results indicate that the mere presence of sarcolemma, and LTCC, may play a role.

Indeed, presence and activation of LTCC per se can enhance RyR activity even without enhanced Ca^2+^ influx, as shown by the effects of BayK in rat ventricular myocytes [26]. Further evidence comes from studies on the development of spark activity in fetal and neonatal cardiac cells which are devoid of TTs. Sparks are at first absent but appear once membrane invaginations develop and LTCCs colocalize with RyRs [27]. Initially, this occurs predominantly near the sarcolemmal membrane [27], [28].

Alternatively, RyRs next to TT membrane may be exposed to elevated local [Ca^2+^] during excitation-contraction coupling which can lead to activation of CaMKII in the dyad [29], [30]. This would result in enhanced activation of RyR near TT.

Local differences in SR Ca^2+^ content are unlikely to underlie the observed differences. Zima et al. [31] showed that not all release sites re-fill at the same rate after sparks, indicating that temporarily gradients may exist, but these are short lived (<100 ms). Further study, using intra-SR low affinity Ca^2+^ probes is needed to investigate the possible existence of standing gradients in the SR. However, earlier data on caffeine-induced release in different areas suggest that there are no differences in local SR Ca^2+^ availability on the timeframe of the measurements carried out in the present study [12].

### Regulation of spark duration by NCX

Detubulation in rat ventricular cells prolonged sparks, indicating a role for sarcolemmal Ca^2+^ flux in modulation of spark duration [11]. Blocking NCX indeed prolonged sparks specifically in early areas. The shorter duration could also result from a locally higher SERCA activity [32] as a higher local Ca^2+^ in the dyad could activate SERCA to a larger extent in near-TT sparks. However, the observation that spark duration was similar in all areas after Ni^2+^ argues against this hypothesis.

The mechanism of modulation of spark duration by NCX is at present not resolved. A straightforward interpretation is a direct modulation of local Ca^2+^ by NCX as Ca^2+^ removal system. Trafford et al. [33] showed that spontaneous Ca^2+^ waves and caffeine-elicited NCX currents had two components, of which they attributed the first part to near-sarcolemmal, dyadic, release which rapidly and locally activated NCX. Recent data from our group on [Ca^2+^] near release sites indicates that a fraction of NCX indeed senses high local [Ca^2+^] during triggered SR Ca^2+^ release and can thus contribute significantly to local Ca^2+^ removal [34]. Similarly, NCX can be activated locally by junctional Ca^2+^ sparks and thus reduce spark duration specifically in areas close to TTs. In contrast, some previous studies have shown that (global) spark duration was not affected by NCX inhibition in rat ventricular myocytes [32], [35]. This effect might be species-dependent, with a relatively low dependence of Ca^2+^ removal on NCX in rat compared to larger mammals [36].

An alternative explanation is that the local removal of Ca^2+^ by NCX reduces the duration of RyR openings. Modulation of RyR gating by NCX has been studied mainly with regard to the probability of opening. Several studies support the idea that reverse mode NCX during depolarization provides an additional trigger promoting RyR opening [37], [38]. In the current study where sparks occur at resting membrane potential, NCX will predominantly act in forward mode. If NCX acts through an effect on gating, one could expect an additional effect of NCX block on frequency of sparks. There was indeed a trend to higher frequency, but this was not significant.

Taken together, NCX modulates spark duration near TTs most likely directly through increased removal of local Ca^2+^ with or without an additional indirect modulation of RyR gating.

### Effect of cellular remodeling in the area adjacent to MI on near-TT and non-coupled sparks

Global spark mass and calculated spark-mediated leak were increased significantly in MI. Whereas at first this feature seems to compare to observations in non-ischemic heart failure models [39], [40] or the changes in the rat with heart failure after MI [41], further analysis shows several differences. Unlike in heart failure models, the global increase in spark mass was not primarily mediated through an increase in average spark frequency. Increase in spark mass was primarily due to a higher average amount of Ca^2+^ released per spark with larger width and amplitude. These data are consistent with the increased SR Ca^2+^ content, a feature not observed in heart failure. The relation between an increase of SR Ca^2+^ content and increased spark size [42] or total SR Ca^2+^ leak [22] is well-established. For a similar increase in SR Ca^2+^ content, leak measured as increase in cytosolic [Ca^2+^] in normal rabbit myocytes, increased two-threefold [22], i.e. in the same range as the present data. While this analysis suggests that the increase in SR content in MI can account for the higher leak, more explicit measurements of leak are needed. Indeed, recent data suggest that a substantial leak can occur without visible sparks [43], [44].

Further subcellular analysis shows heterogeneity in the changes in local spark properties. Such heterogeneity in local Ca^2+^ release was also described in the dog with pacing-induced heart failure [17]. In this model, the loss of TTs led to areas with reduced sparks and a larger beat-to-beat spark variance. In the present data, an increase in frequency was seen in delayed areas, though not in early areas. The former is consistent with the expectation of increased frequency related to a higher SR Ca^2+^ content. The latter, the absence of increase in early release sites, could be related to changes in the dyadic environment, such as a reduction in the amount of L-type Ca^2+^ channels per couplon [18], a change in the dyadic space geometry, or a reduction in RyR P_o_, which may offset the effect of an increased SR Ca^2+^ content. This needs further investigation.

**Figure 7:**
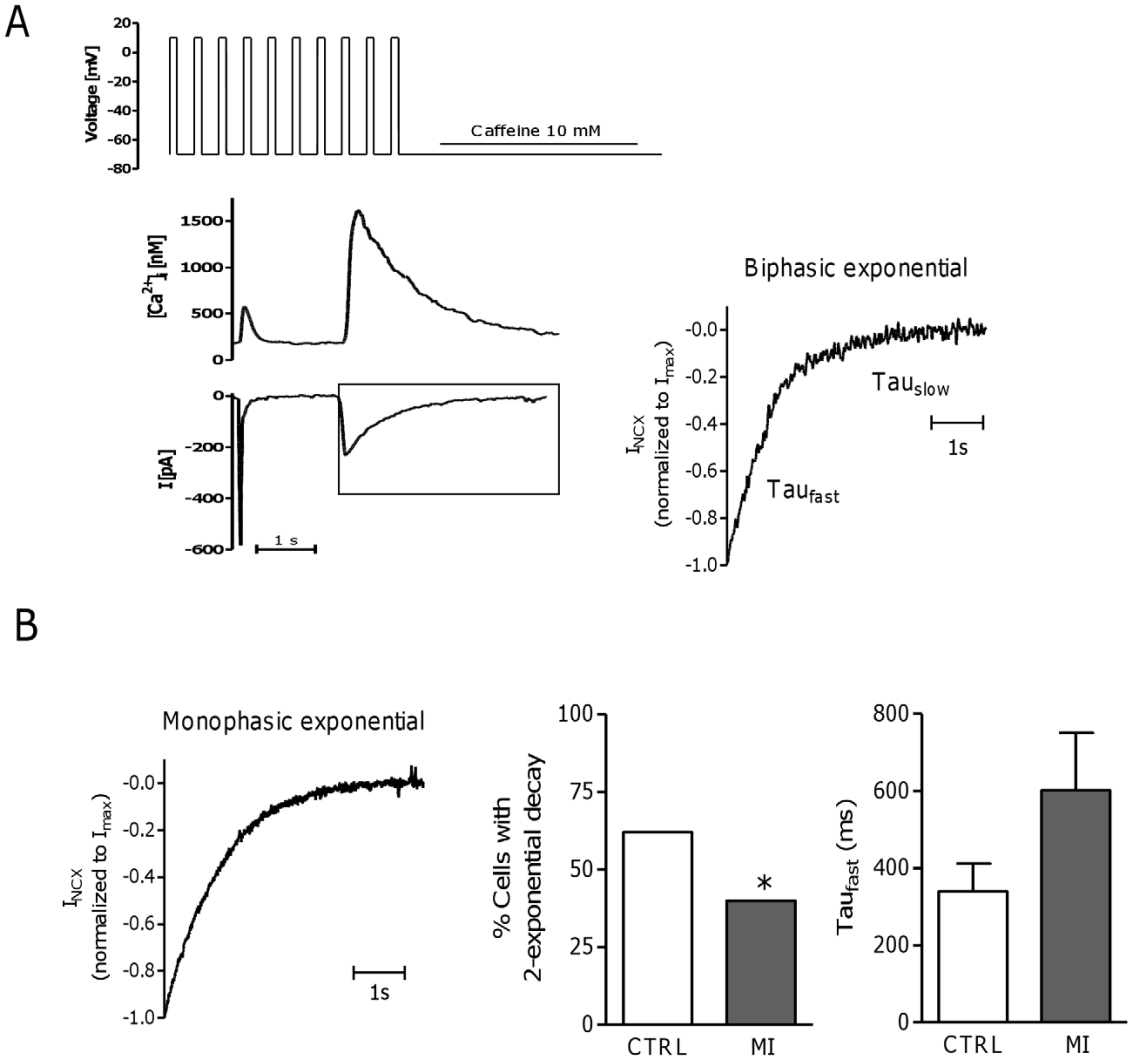
Ca^2+^ removal by NCX during caffeine application. A. Example of current and [Ca^2+^]_i_ transient recording obtained during the last conditioning pulse from -70 to +10 mV and caffeine application (left). The decay of the current was fit by a 1- or 2-exponential according to the goodness of fit (R^2^>0.95) and with the 2 amplitudes being negative. The right panel is a typical example of a 2-exponential decay in a CTRL myocyte. B. Example of monophasic I_NCX_ decay (left) and mean data on incidence of biphasic decay (middle). The percentage of cells better fit by a biphasic exponential was significantly higher in CTRL than in MI (CTRL, N_pigs_ = 4, n_cells_ = 17; MI, N_pigs_ = 4, n_cells_ = 12, P<0.05). In addition, Tau of fast component of I_NCX_ decay tended to be faster in CTRL than in MI (right, CTRL, N_pigs_ = 4, n_cells_ = 8, *vs*. MI, N_pigs_ = 2, n_cells_ = 4, P = NS). * denotes P<0.05.

Spark duration increased preferentially near TTs in MI, suggesting that local NCX Ca^2+^ removal is reduced. The reduced NCX current density is consistent with this hypothesis, which is further supported by analysis of the phases of Ca^2+^ removal during caffeine application (Fig. 7) [33]. In the majority of CTRL cells (62%), two clear phases of NCX mediated efflux were evident (Fig.7A). In MI only 40% showed this type of release, with a trend to a longer Tau_fast_ (Fig.7B), indicating a smaller component of NCX Ca^2+^ removal from junctional release sites, and thus a reduction of T-tubular NCX. These results suggest a redistribution of NCX transporters between the TT and other sarcolemmal compartments, since global NCX expression remained unchanged after MI [18].

Interestingly, an increase in the amount of cells with long sparks was observed in MI. Since the occurrence of long sparks was not different between the types of release areas, the mechanism for long sparks is probably different from the NCX-mediated regulation of spark duration described above. Long sparks are most likely related to altered gating properties of RyR clusters. These have been reported earlier to correlate with RyR subconductance states [45] and with downregulation of FK506-binding proteins FKBP12 and FKBP12.6 [46]. Computational modeling has shown that when inter-RyR coupling is decreased spark duration is prolonged [47]. Pharmacological reduction of RyR P_o_ in combination with increased SR load leads to prolonged spark events [48]. In MI the increased SR Ca^2+^ load may enhance the occurrence of long sparks, although changes in RyR regulation may also contribute.

### Perspectives for myocyte function after MI

The current data were obtained from pigs with a moderate size infarct in the posterior wall that did not lead to global heart failure; myocytes were obtained from an area with reduced perfusion adjacent to the MI [18]. This model represents a stage and aspect of ischemic cardiomyopathy in which NCX is not upregulated and SR content not reduced, as typically described in models of heart failure. It should be noted that NCX upregulation is not a universal feature of cardiac remodeling [49]. The combination of loss of TTs, high SR content and low NCX set the stage for a specific myocyte phenotype.

The redistribution of spark sites with a reduced fraction of sites near-TT will by itself reduce the extrusion of released Ca^2+^ from the cell by NCX. Indeed, at delayed release areas, which represent the largest fraction of the cell, spark frequency is increased but the distance to NCX reduces Ca^2+^ removal. In addition, at early sites, NCX density is reduced, so cellular Ca^2+^ loss will be small. Therefore, although total spark mass is increased, actual spark-mediated cellular Ca^2+^ efflux is minimized. This mechanism would preserve SR Ca^2+^ content.

Reduced removal of spontaneous Ca^2+^ release and higher SR Ca^2+^ content may on the other hand promote transition from sparks to waves and therefore the potential for arrhythmias. In the 6-week follow-up of the animals, mortality is very low and does not allow conclusions regarding arrhythmias but some information can be gained from the cellular data. In cells that showed spontaneous Ca^2+^ waves, the time to the first wave (measured from the end of the last conditioning transient) was significantly shorter in MI (8.6±1.7 s in MI, n_cells_ = 22, vs. 12.5±1.6 s in CTRL, n_cells_ = 23, p<0.05) and the frequency of wave activity was higher in MI cells (0.15±0.03 waves/s in MI, vs. 0.09±0.05 waves in CTRL). These results are in line with an increased tendency for wave initiation and propagation in MI cells. A reduced density of NCX may however mitigate the arrhythmogenic potential of such waves.

### Conclusions

In ventricular myocytes with a sparse TT network, RyR cluster properties related to their position relative to TTs lead to intracellular heterogeneity of sparks. Remodeling in the area adjacent to a moderate MI differentially affects RyR subpopulations. Loss of TTs leads to a smaller fraction of RyR near TTs. Redistribution of sparks away from the membrane together with reduced NCX in TTs may limit actual Ca^2+^ loss from the cell and contribute to the increased SR Ca^2+^ content. The increased spark mass and SR content can increase the propensity for spontaneous Ca^2+^ wave generation but the lower density of NCX may mitigate the arrhythmogenic potential.

## References

[1] Franzini-Armstrong C, Protasi F, Ramesh V (1999). Shape, size, and distribution of Ca^2+^ release units and couplons in skeletal and cardiac muscles.. Biophys J.

[2] Scriven DR, Dan P, Moore ED (2000). Distribution of proteins implicated in excitation-contraction coupling in rat ventricular myocytes.. Biophys J.

[3] Cheng H, Lederer WJ, Cannell MB (1993). Calcium sparks: elementary events underlying excitation-contraction coupling in heart muscle.. Science.

[4] Lopez-Lopez JR, Shacklock PS, Balke CW, Wier WG (1995). Local calcium transients triggered by single L-type calcium channel currents in cardiac cells.. Science.

[5] Cleemann L, Wang W, Morad M (1998). Two-dimensional confocal images of organization, density, and gating of focal Ca^2+^ release sites in rat cardiac myocytes.. Proc Natl Acad Sci U S A.

[6] Shacklock PS, Wier WG, Balke CW (1995). Local Ca2+ transients (Ca2+ sparks) originate at transverse tubules in rat heart cells.. J Physiol (Lond).

[7] Huser J, Lipsius SL, Blatter LA (1996). Calcium gradients during excitation-contraction coupling in cat atrial myocytes.. J Physiol (Lond).

[8] Cordeiro JM, Spitzer KW, Giles WR, Ershler PE, Cannell MB (2001). Location of the initiation site of calcium transients and sparks in rabbit heart Purkinje cells.. J Physiol (Lond).

[9] Lipp P, Huser J, Pott L, Niggli E (1996). Spatially non-uniform Ca2+ signals induced by the reduction of transverse tubules in citrate-loaded guinea-pig ventricular myocytes in culture.. J Physiol (Lond).

[10] Louch WE, Bito V, Heinzel FR, Macianskiene R, Vanhaecke J (2004). Reduced synchrony of Ca^2+^ release with loss of T-tubules - a comparison to human failing cardiac myocytes.. Cardiovasc Res.

[11] Brette F, Despa S, Bers DM, Orchard CH (2005). Spatiotemporal characteristics of SR Ca(2+) uptake and release in detubulated rat ventricular myocytes.. J Mol Cell Cardiol.

[12] Heinzel FR, Bito V, Volders PG, Antoons G, Mubagwa K (2002). Spatial and temporal inhomogeneities during Ca2+ release from the sarcoplasmic reticulum in pig ventricular myocytes.. Circ Res.

[13] Crossman DJ, Ruygrok PN, Soeller C, Cannell MB (2011). Changes in the organization of excitation-contraction coupling structures in failing human heart.. PLoS One.

[14] Lukyanenko V, Ziman A, Lukyanenko A, Salnikov V, Lederer WJ (2007). Functional groups of ryanodine receptors in rat ventricular cells.. J Physiol.

[15] Sheehan KA, Zima AV, Blatter LA (2006). Regional differences in spontaneous Ca2+ spark activity and regulation in cat atrial myocytes.. J Physiol.

[16] Woo SH, Cleemann L, Morad M (2003). Spatiotemporal characteristics of junctional and nonjunctional focal Ca2+ release in rat atrial myocytes.. Circ Res.

[17] Meethal SV, Potter KT, Redon D, Munoz-del-Rio A, Kamp TJ (2007). Structure-function relationships of Ca spark activity in normal and failing cardiac myocytes as revealed by flash photography.. Cell Calcium.

[18] Heinzel FR, Bito V, Biesmans L, Wu M, Detre E (2008). Remodeling of T-tubules and reduced synchrony of Ca^2+^ release in myocytes from chronically ischemic myocardium.. Circ Res.

[19] Volkers M, Loughrey CM, Macquaide N, Remppis A, DeGeorge BR (2007). S100A1 decreases calcium spark frequency and alters their spatial characteristics in permeabilized adult ventricular cardiomyocytes.. Cell Calcium.

[20] Cheng H, Song LS, Shirokova N, Gonzalez A, Lakatta EG (1999). Amplitude distribution of calcium sparks in confocal images: theory and studies with an automatic detection method.. Biophys J.

[21] Despa S, Brette F, Orchard CH, Bers DM (2003). Na/Ca exchange and Na/K-ATPase function are equally concentrated in transverse tubules of rat ventricular myocytes.. Biophys J.

[22] Shannon TR, Ginsburg KS, Bers DM (2002). Quantitative assessment of the SR Ca2+ leak-load relationship.. Circ Res.

[23] Satoh H, Blatter LA, Bers DM (1997). Effects of [Ca2+]i, SR Ca2+ load, and rest on Ca2+ spark frequency in ventricular myocytes.. Am J Physiol.

[24] Mackenzie L, Bootman MD, Berridge MJ, Lipp P (2001). Predetermined recruitment of calcium release sites underlies excitation-contraction coupling in rat atrial myocytes.. J Physiol.

[25] Woo SH, Cleemann L, Morad M (2003). Spatiotemporal characteristics of junctional and nonjunctional focal Ca2+ release in rat atrial myocytes.. Circ Res.

[26] Katoh H, Schlotthauer K, Bers DM (2000). Transmission of information from cardiac dihydropyridine receptor to ryanodine receptor: evidence from BayK 8644 effects on resting Ca(2+) sparks.. Circ Res.

[27] Seki S, Nagashima M, Yamada Y, Tsutsuura M, Kobayashi T (2003). Fetal and postnatal development of Ca2+ transients and Ca2+ sparks in rat cardiomyocytes.. Cardiovasc Res.

[28] Haddock PS, Coetzee WA, Cho E, Porter L, Katoh H (1999). Subcellular [Ca2+]i gradients during excitation-contraction coupling in newborn rabbit ventricular myocytes.. Circ Res.

[29] Song Q, Saucerman JJ, Bossuyt J, Bers DM (2008). Differential integration of Ca2+-calmodulin signal in intact ventricular myocytes at low and high affinity Ca2+-calmodulin targets.. J Biol Chem.

[30] Saucerman JJ, Bers DM (2008). Calmodulin mediates differential sensitivity of CaMKII and calcineurin to local Ca2+ in cardiac myocytes.. Biophys J.

[31] Zima AV, Picht E, Bers DM, Blatter LA (2008). Termination of cardiac Ca2+ sparks: role of intra-SR [Ca2+], release flux, and intra-SR Ca2+ diffusion.. Circ Res.

[32] Gomez AM, Cheng H, Lederer WJ, Bers DM (1996). Ca2+ diffusion and sarcoplasmic reticulum transport both contribute to [Ca2+]i decline during Ca2+ sparks in rat ventricular myocytes.. J Physiol.

[33] Trafford AW, Diaz ME, O'Neill SC, Eisner DA (1995). Comparison of subsarcolemmal and bulk calcium concentration during spontaneous calcium release in rat ventricular myocytes.. J Physiol (Lond).

[34] Acsai K, Antoons G, Livshitz L, Rudy Y, Sipido KR (2011). Microdomain [Ca(2)] near ryanodine receptors as reported by L-type Ca(2) and Na+/Ca(2) exchange currents.. J Physiol.

[35] Goldhaber JI, Lamp ST, Walter DO, Garfinkel A, Fukumoto GH (1999). Local regulation of the threshold for calcium sparks in rat ventricular myocytes: role of sodium-calcium exchange.. J Physiol (Lond).

[36] Bassani JW, Bassani RA, Bers DM (1994). Relaxation in rabbit and rat cardiac cells: species-dependent differences in cellular mechanisms.. J Physiol (Lond).

[37] Neco P, Rose B, Huynh N, Zhang R, Bridge JH (2010). Sodium-calcium exchange is essential for effective triggering of calcium release in mouse heart.. Biophys J.

[38] Sobie EA, Cannell MB, Bridge JH (2008). Allosteric activation of Na^+^-Ca^2+^ exchange by L-type Ca^2+^ current augments the trigger flux for SR Ca^2+^ release in ventricular myocytes.. Biophys J.

[39] Shannon TR, Pogwizd SM, Bers DM (2003). Elevated sarcoplasmic reticulum Ca2+ leak in intact ventricular myocytes from rabbits in heart failure.. Circ Res.

[40] Toischer K, Rokita AG, Unsold B, Zhu W, Kararigas G (2010). Differential cardiac remodeling in preload versus afterload.. Circulation.

[41] Lyon AR, MacLeod KT, Zhang Y, Garcia E, Kanda GK (2009). Loss of T-tubules and other changes to surface topography in ventricular myocytes from failing human and rat heart.. Proc Natl Acad Sci U S A.

[42] Lukyanenko V, Viatchenko-Karpinski S, Smirnov A, Wiesner TF, Gyorke S (2001). Dynamic regulation of sarcoplasmic reticulum Ca(2+) content and release by luminal Ca(2+)-sensitive leak in rat ventricular myocytes.. Biophys J.

[43] Santiago DJ, Curran JW, Bers DM, Lederer WJ, Stern MD (2010). Ca sparks do not explain all ryanodine receptor-mediated SR Ca leak in mouse ventricular myocytes.. Biophys J.

[44] Zima AV, Bovo E, Bers DM, Blatter LA (2010). Ca(2)+ spark-dependent and -independent sarcoplasmic reticulum Ca(2)+ leak in normal and failing rabbit ventricular myocytes.. J Physiol.

[45] Xiao RP, Valdivia HH, Bogdanov K, Valdivia C, Lakatta EG (1997). The immunophilin FK506-binding protein modulates Ca2+ release channel closure in rat heart.. J Physiol.

[46] Gomez AM, Rueda A, Sainte-Marie Y, Pereira L, Zissimopoulos S (2009). Mineralocorticoid modulation of cardiac ryanodine receptor activity is associated with downregulation of FK506-binding proteins.. Circulation.

[47] Sobie EA, Dilly KW, dos Santos CJ, Lederer WJ, Jafri MS (2002). Termination of cardiac Ca(2+) sparks: an investigative mathematical model of calcium-induced calcium release.. Biophys J.

[48] Zima AV, Picht E, Bers DM, Blatter LA (2008). Partial inhibition of sarcoplasmic reticulum ca release evokes long-lasting ca release events in ventricular myocytes: role of luminal ca in termination of ca release.. Biophys J.

[49] Sipido KR, Volders PG, Vos MA, Verdonck F (2002). Altered Na/Ca exchange activity in cardiac hypertrophy and heart failure: a new target for therapy?. Cardiovasc Res.

